# Hiding Behind Machines: Artificial Agents May Help to Evade Punishment

**DOI:** 10.1007/s11948-022-00372-7

**Published:** 2022-04-04

**Authors:** Till Feier, Jan Gogoll, Matthias Uhl

**Affiliations:** 1 TUM School of Governance, TU Munich, Richard-Wagner-Straße 1, 80333 Munich, Germany; 2 Bavarian Institute for Digital Transformation, TU Munich, Gabelsbergerstr. 4, 80333 Munich, Germany; 3 Faculty of Computer Science, Technische Hochschule Ingolstadt, Esplanade 10, 85049 Ingolstadt, Germany

**Keywords:** Automation, Ethics, Experiment, Responsibility, Algorithm

## Abstract

The transfer of tasks with sometimes far-reaching implications to autonomous systems raises a number of ethical questions. In addition to fundamental questions about the moral agency of these systems, behavioral issues arise. We investigate the empirically accessible question of whether the imposition of harm by an agent is systematically judged differently when the agent is artificial and not human. The results of a laboratory experiment suggest that decision-makers can actually avoid punishment more easily by delegating to machines than by delegating to other people. Our results imply that the availability of artificial agents could provide stronger incentives for decision-makers to delegate sensitive decisions.

## Introduction

In this study, we investigate whether delegators are able to successfully avoid punishment, or at least mitigate it, by delegating tasks to artificial agents. Our investigation focuses on whether people judge delegations to human and artificial agents differently in light of given outcomes. In particular, by analyzing the respective punishment patterns, we hope to learn whether the introduction of artificial agents provides an incentive for strategic scapegoating, i.e., successfully shifting punishment to the artificial agent.

At the core of this study is a laboratory experiment in which participants had to make a number of decisions that would affect their own payment as well as the payment of other subjects. The two main components were a delegation decision and a stage during which participants could punish and reward other subjects.

The delegation decision consisted of two parts. First, subjects had to complete a number of logic puzzles in a given time frame, knowing that this task would affect the payment for participation in the experiment. Second, they were presented with a choice: They could either make their own performance count or rely on the performance of an agent instead. This agent was a randomly matched player in the baseline treatment and an algorithm acting as a non-human agent in the manipulation. To add a normative component to the experiment, we made it affect not the delegator’s own payment but the payment of another player. A poor decision could therefore cause real monetary harm to a third party. Participants were later given the opportunity to punish or reward each other based on the decision to delegate or not and the respective outcomes. They could do so by increasing or reducing the payment of the randomly matched player whose decision had an impact on them. Participants were therefore given an option to express their disapproval in a quantifiable way.

We found no differences between judgments toward human and artificial agents in the event of good outcomes. This means that the beneficial delegation to an artificial agent was considered neither better nor worse than the beneficial delegation to a human agent. However, when negative outcomes occurred, delegators fared significantly better if a machine agent caused the failure. Interestingly, participants did not seem to anticipate this pattern as we did not find significant differences regarding delegation decisions themselves. In fact, decisions involving human and artificial agents seem to be driven by the expected utility of the delegation for the affected party.

Our article proceeds as follows. In Section “Blame Avoidance”, we conceptualize our use of the term blame and justify its operationalization through the measurement of punishment. We then briefly discuss the value of empirical research for the ethical debate on blame avoidance and derive our research question. In Section “Experiment Design”, we outline the experimental setup we developed to test our research question. We discuss our findings in Section “Results” and conclude in the final section.

## Blame Avoidance

### The Concept of Blame and the Role of Empirical Research

Normative ethics raises a fundamental question with respect to the increasing use of artificial agents in decision-making: In which sense can artificial agents be held responsible for their actions? Philosophers and engineers started pondering this question decades ago when computers merely functioned as calculators (Moor, 1979). There is still little consensus on the matter, and the idea of “moral machines” remains under debate (Allen & Wallach, 2012).

One’s willingness to ascribe moral responsibility to an agent might largely depend on our definition of blame (Smith, 2013). Blame is an extraordinarily rich concept (Malle et al., 2014) and there are several philosophical accounts of the concept, which each stress different aspects and functions (Coates & Tognazzini, 2013). At its most general level, it is understood as “a reaction to something of negative normative significance about someone or their behavior” (Tognazzini & Coates, 2018). In our study, we used punishment as a behavioral proxy for attributing blame. This entails that our findings alone are not necessarily of moral significance. Retributive acts can be meted out without ethical justification or any regard for morality and we do not know our participants' considerations leading to their decisions. However, we do believe that our findings have relevance for the ethical debate on algorithms because the possibility to deflect punishment through the delegation to algorithms may foster their potential misuse. This reveals an important advantage of our approach: blame is most relevant when it is operationalized into punishment (von Grundherr et al., 2021). The mere experience of being blamed is less likely to make wrongdoers adapt their behavior, and actual punishment has a much stronger deterrence effect (Klepper & Nagin, 1989). Put differently, as we are looking for behavioral factors that are relevant to the ethical debate, it seems meaningful to focus on the most potent ones. A better understanding of how people actually interact with machines and how they judge the respective interactions could also provide valuable empirical feedback for the formulation of ethical guidelines.

This aspect is closely linked to another issue, the so-called retribution gap, first introduced by Danaher, (2016). Danaher argues that humans are innate retributivists who seek to identify and punish culpable wrongdoers whenever they perceive harm. This becomes problematic when harm is brought about by robots or other non-human agents. An example is the crash of a self-driving car during which people are injured or killed (Nyholm, 2018). While Malle et al., (2019) provide evidence that people seek to apply similar norms to human and artificial agents, it may prove difficult to translate blame placed on artificial agents into punishment, leaving no appropriate object for the desired retributive punishment. According to Danaher, this is not a problem only for deontologists or moral retributionists, i.e., those who believe that people should be punished because it is intrinsically just. Potential problems include an increased risk of moral scapegoating as well as a potential threat to the rule of law.

Real-life examples of this were demonstrated in the aftermath of the death of Elaine Herzberg, a woman killed by a self-driving car in Arizona in 2018. Arizonans attacked self-driving cars and harassed their passengers who had no connection to the aforementioned accident (Romero, 2018). This behavior could be an indicator that the retribution gap does, in fact, exist and affect behavior. A better understanding of when and how retribution gaps arise could help to prevent such events in the future. Their empirical and psychological relevance is not dependent on whether one believes that retributivist intuitions have any normative significance. Kraaijeveld (2019), for instance, argues that retributive intuitions cannot justify retribution in cases of harm brought about by non-human agents as they are not eligible targets for moral blame. The crucial task would thus become to exercise control over retributive intuitions and to make sure that we do not engage in unjust recrimination. We believe that a better empirical understanding of the related intuitions is essential to this endeavor.

As already mentioned above, we believe that empirical research may be informative for the debate on blame in the context of artificial agents. Normativity relies on prescriptive arguments based on reasons rather than descriptive analysis of behavior. While this is certainly true, it seems rather obvious that normative arguments are informed by empirical facts. No other than Kant (2003) acknowledges this when he claims that “ought implies can.” To give a prominent example: Rawls’ (1971) Theory of Justice relies heavily on empirical observations. First, his concept of a personal reflective equilibrium relies on introspective evidence—a method that Güth and Kliemt (2010) consider to be a form of “armchair empiricism.” Second, Rawls specifically uses insights from economics and psychology to justify his difference principle because it relies on the empirical assumption that people are, on average, risk averse (Rawls, 1971). The question of whether people are, in fact, risk averse is certainly not an a priori assumption but is the outcome of observation and experiments. While philosophers have been influenced by the findings of other, mostly empirical, sciences, there is also the rather new field of experimental philosophy. Interesting insights have been generated regarding ethical issues like intentional actions and relevant side effects (Knobe, 2003). Kraaijeveld (2021) argues that despite the empirical turn that the philosophy of technology has taken, experimental philosophy has received virtually no attention in the realm of the philosophy of technology. He advocates extending the systematic investigation of people’s intuitions, which has already provided inspiring insights in other domains of philosophy, to questions related to the philosophy of technology.

## Previous Studies and Aim of Present Study

A substantial amount of literature is available on the parameters that influence automation use in teams of human supervisors and the machines at their disposal (Dzindolet et al., 2002). A recurring phenomenon in this context is machine aversion, i.e., people’s aversion to machine use regardless of the machine's capabilities. In some instances, people even prefer humans over algorithms after having seen the latter outperforming their human counterparts (Dietvorst et al., 2015).

In recent years, a number of studies investigated how this effect changes when decisions have moral implications. In these studies, a decision was considered morally relevant if it imposed indirect costs or benefits to an uninvolved third party. In this sense, a decision could only be selfish or fair with respect to others. Goldbach et al. (2019) found that people were hesitant to delegate decisions to algorithms when the decision affected both the decision-maker and a third party. Similarly, a study by Niszczota and Kaszás (2020) suggests that algorithm aversion extends to the financial sector and that people especially prefer human over artificial agents when it comes to making financial decisions with moral implications. In a laboratory study, Gogoll and Uhl (2018) identified a strong aversion against delegating other-regarding tasks to algorithms. It seems that people were less willing to delegate tasks to machines if those decisions imposed monetary externalities on third parties. While their study assessed the “perceived utility” of the artificial agent and the trust in that agent, they were unable to determine the exact causes of the profound algorithm aversion.

Other studies also support the idea that a lack of trust is unlikely to be the cause of aversion towards machine use. If anything, there seems to be an over-reliance on and over-trust in machines, even if the lives of people are at stake (Robinette et al., 2016). This suggests that there must be other causes for machine aversion in decisions with moral implications. We hypothesize that the avoidance of punishment or blame-shifting are key concepts in understanding this phenomenon. Perceived responsibility is already an important research topic in relation to machine use (Hevelke & Nida-Rümelin, 2015). However, little attention has been paid to the question of how the introduction of machine agents might affect the blame and praise that people ascribe to the delegator in light of a given outcome. Understanding this would be an important step in understanding how the availability of artificial agents might influence people’s motivation to delegate morally sensible tasks. This is closely linked to the idea that avoiding punishment may be a pivotal factor regarding decisions to delegate in general.

Strategies of blame avoidance have long been discussed in the political sciences (Weaver, 1986). Instances of so-called blame games can frequently be observed in political systems with regard to policymaking and implementation in the European Union (Heinkelmann-Wild & Zangl, 2020) or between officials from different levels of government in the United States (Maestas et al., 2008). Unsurprisingly, similar strategies can also be observed in the private sector, for instance, when it comes to upholding employment standards within franchise networks (Hardy, 2019). The idea that blame shifting can, in fact, be a pivotal factor regarding delegation decisions plays an especially prominent role in public choice theory (Fiorina, 1986).

Experimental evidence of this phenomenon comes from Fischbacher et al. (2008), who showed that the attribution of blame can sometimes be effectively shifted and that this constitutes a powerful motive for decision-makers. Other experiments provide evidence that this is even true if the blamed delegate was effectively powerless (Hill, 2015) or if the delegation decision itself eliminated the possibility of a moral outcome (Oexl & Grossman, 2013). But does this also hold true for artificial agents?

Popular culture assigns a high degree of importance to human accountability, despite an empirical decline of human control in many areas (Elish & Hwang, 2015). It appears that artificial agents are faulted less for errors and wrongdoings than human agents. This is sometimes referred to as “algorithmic outrage asymmetry.” This concept suggests that people are less outraged by algorithmic wrongdoing, for instance, in cases of discrimination by age, race, or gender than by human wrongdoing (Bigman et al., 2020). This is supported by empirical evidence which shows that moral attributions are generally weakened for artificial agents (Gamez et al., 2020). Thus, some researchers fear that humans could emerge as “moral crumple zones” and would have to take on blame even for accidents outside of their control (Elish, 2019).

While some argue that humans will bear all of the responsibility and none of the control when working with machines, others think that machines are perfectly suited to be used as scapegoats, especially if they show signs of situational awareness, free will, and intentionality. Bigman et al. (2019) and Shank, DeSanti, et al. (2019), Shank, Graves, et al. (2019) report that while people attribute less fault to AI for moral wrongdoing, humans who monitor AI are also faulted less than humans who work solo, i.e., in human-only teams. However, the results are ambiguous. Strobel and Kirchkamp (2017), for instance, investigated whether choices and perceived guilt in a dictator game change when players share responsibility with machines. The authors reported that perceived responsibility and guilt did not vary significantly between human–human teams and human–machine teams. They did notice, however, that people tended to make fewer selfish decisions when partnered with machines, although that effect was statistically insignificant.

So, while blame avoidance has long been established as an integral part of delegation decisions, empirical evidence of whether the introduction of artificial agents is rendering this motive less or more important is lacking. This constitutes a serious research gap, the implications of which extend beyond academia. A better understanding of blame-shifting to artificial agents could explain over- and under-reliance on machines and profoundly influence legal decision-making regarding automation and digitization. For instance, administrators are struggling to provide governance strategies for automated vehicles because of the ambiguity with respect to liability (Taeihagh & Lim, 2019). A better understanding of how people actually attribute blame would be helpful in creating guidelines that are not only more effective but also more likely to gain consensus.

Deeper insights into the phenomenon might also help to shield human operators from unjust recrimination. As mentioned above, our classic understanding of human–machine teams has cemented a focus on human responsibility despite a decline of human control in various areas (Elish & Hwang, 2015). This is especially troubling since responsibility has proven to be an important factor in understanding and predicting punishment patterns (Bolton & Ockenfels, 2000; Fehr & Schmidt, 1999). It seems that human operators are, in fact, in harm’s way and might become scapegoats in cases of technical failure.

In contrast, machine use might emerge as a strategy for self-exculpation in critical situations. This could have detrimental effects if it leads to an overuse of machines—a bleak prospect given the growing capabilities of algorithms and the potential harm this implies for workers and consumers. In sum, there are plenty of reasons to investigate punishment patterns and, eventually, attributions of blame in the context of automation.

To shed light on this problem, we tested the following conjecture in a laboratory experiment.

**Conjecture**: Delegators are rewarded differently for delegating other-regarding tasks to artificial agents as compared to human agents.

On one hand, the delegation to an artificial agent of a task that could carry severe consequences for a third party might be considered careless and result in punishment or defamation. On the other hand, principals might be exculpated entirely since people deem the failure of machines to be more outside of the principals’ control than the failure of another human to whom they delegated the task. Either way, a better understanding of public reservations regarding the introduction of novel technology is likely to prove useful in future moral and legal considerations. The experiment designed to test the above conjecture is outlined in the following section.

Additionally, we explored whether the effect of perceived utility on delegation decisions varies depending on the agent’s artificial or human nature. Based on a definition by Dzindolet et al. (2002), we define the perceived utility of employing an artificial agent as the difference between the perceived reliability of an automated device and the perceived reliability of manual control. Furthermore, we incorporated risk attitudes into our analysis as a control (O’Donoghue & Somerville, 2018).

## Experiment Design

The experiment consisted of (1) a logic task, (2) a delegation decision, (3) an evaluation of the delegation decision, (4) a self-assessment of performance, and (5) an elicitation of risk attitudes through choosing a lottery. An experimental currency unit (ECU) was used throughout the experiment with the exchange rate of 10 ECU to 1 EUR. As payment for participation in the experiment, subjects received an initial (guaranteed) 40 ECU participation fee, which was increased by the outcome of the delegation (successful or not), plus (minus) the reward (punishment) for their decision to delegate or not, a bonus for the accuracy of their self-assessment, and the pay-off of the lottery.

Subjects received their instructions on-screen and were fully informed about the rules of the game. They were randomly matched according to a perfect stranger matching, i.e., no two participants interacted more than once during the experiment. The experimental manipulation consisted of changing the nature of the agent to which a task could be delegated: it was either another human participant or an artificial agent (see 3.2). Table 1 gives an overview of the four stages of the experiment.

**Table 1:** Overview of experimental stages

Stage	Human (Machine) treatment
(1) Solve logic task	Participants solve a series of logic puzzles
(2) Make delegation decision	Participants decide whether to delegate to another entity (human or machine) or to have their own work count
(3) Evaluate delegation decision	Participants reward or punish others’ decisions to delegate or not
(4) Self-assess & choose lotteries	Participants guess how many errors they made in the logic task and reveal their risk attitude by choosing between lotteries

### Logic Task

**Fig. 1:**
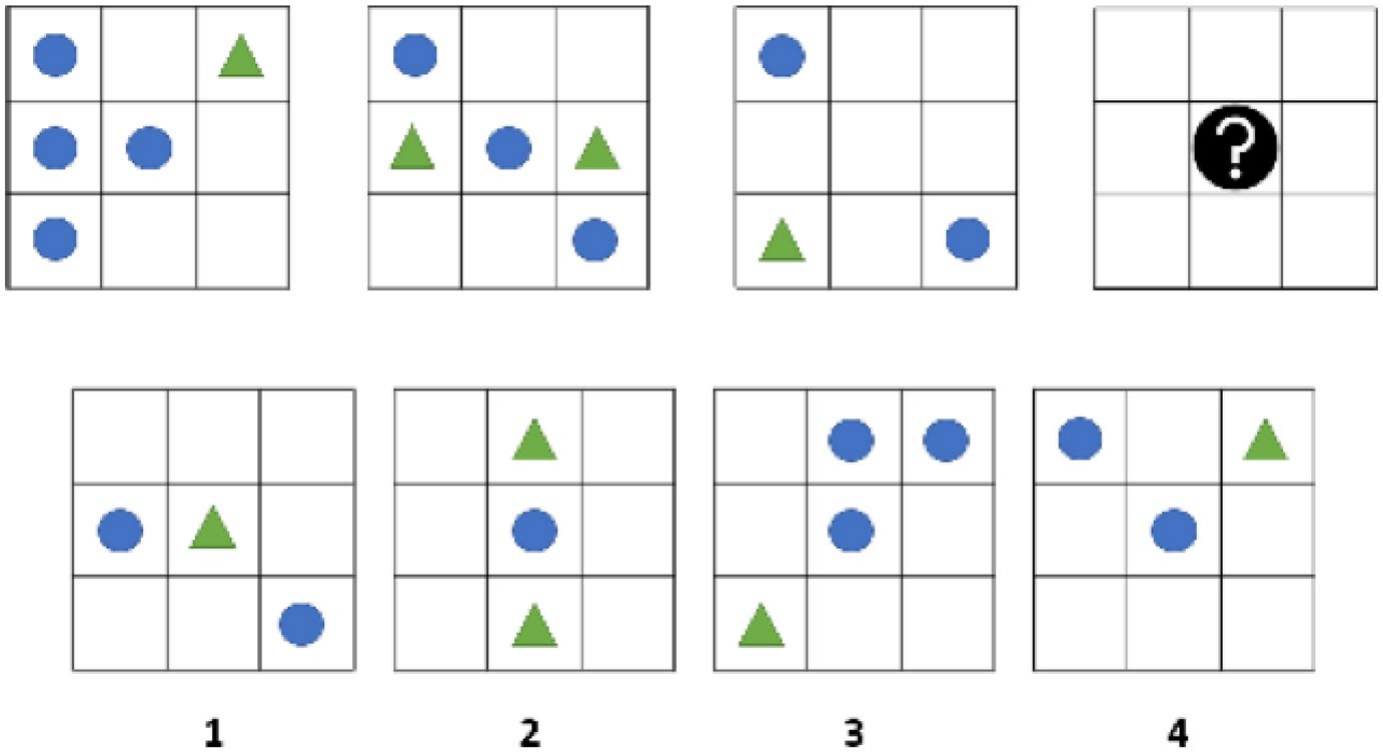
Example of a logic task

The first task of the experiment was a logic puzzle. While this task had no moral relevance per se, it was a necessary pre-stage for the following investigation. We asked participants to complete ten puzzles in a maximum of five minutes. Each puzzle consisted of a sequence of three patterns and a placeholder for the missing fourth pattern. The answer had to be derived from the given sequence and selected from a set of four alternatives. To identify the right answer, participants always had to focus on the circles and ignore other symbols and any colors (see Fig. 1). We assumed that delegation decisions would heavily depend on the agent’s perceived capabilities and therefore chose a task involving visual perception to foster participants’ intuition that the algorithm could err. We did so because the discussion about previous studies indicated that people are skeptical that artificial agents will fail at purely mathematical tasks–an implementation used, for instance, by Gogoll and Uhl (2018). Including an aspect of image recognition, on the other hand, seemed well suited to mitigating the effects of such doubts. Participants had to complete ten of the tasks described above before they could continue to the second stage of the experiment.

### Delegation Decision

**Fig. 2:**
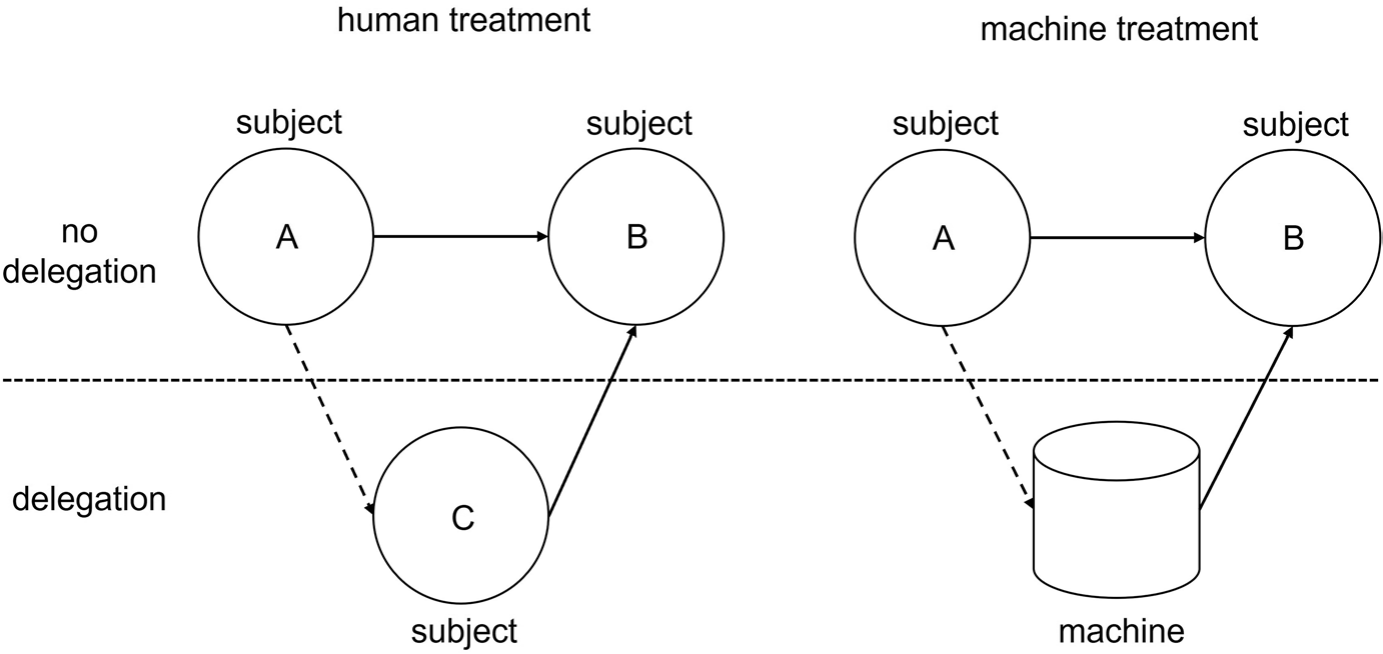
Delegation decision for human and machine treatment

After completing the logic task, participants were randomly assigned to one of two treatments: the “human” or the “machine” treatment. Only at this point did subjects learn the nature of the agent to whom they could delegate the task (see Fig. 2).

To give participants an idea of their potential delegate’s capability, they were shown representations of the agent’s performance. In the human treatment, the participants were shown a histogram displaying the performance of the other participants based on the actual results of the running session. In the machine treatment, participants were shown a histogram with information about how often the algorithm failed to give correct answers in *n* trial runs. The algorithm was programmed to mirror the performance of the human participants in the room. The performance of the artificial agent was, therefore, as good as that of the participants in the respective session. This process ensured that the delegation decisions were based on the agent’s nature instead of any assumptions about differing capabilities. We also ensured that we did not facilitate anthropomorphism because this could have altered our subjects’ subsequent decisions. The algorithm did not have a name, voice, or human-like depiction. Subjects did not interact with the algorithm directly to avoid the impression of a conscious mind. We discuss this decision in more detail in the conclusion of our article.

Participants were then asked to make the delegation decision. They chose whether their own performance or that of their human or artificial agent (depending on the treatment) would determine the third party’s payoff. The delegation decision would thus not affect the payment of the delegator but that of another participant. After participants decided to delegate or not, one of the solutions to the ten puzzles was randomly chosen from their agent’s or their own answers. If the selected solution was correct, the third party received an additional payoff. If the solution was incorrect, the third party did not receive an additional payoff. We argue that this makes our experiment relevant for ethical deliberations. Because we have no reason to believe that a rational participant would prefer less money to more, the decision could do actual, albeit monetary, harm to another person.

### Evaluation of the Delegation

In order to elicit a measure of perceived guilt or responsibility, participants were given the opportunity to punish or reward the participant whose decision to delegate or not affected their own payoff. Participants did not know the actual decision of the participant that they evaluated, nor whether this decision had resulted in a good or bad outcome for themselves. They were asked to increase or decrease the participant’s payment by at most 40 ECU. Reward and punishment choices were contingent on the two possible decisions, to delegate or not, and the two possible outcomes, success or failure. Thus, in all cases, four choices had to be made. Only the adjustment that reflected the participant’s actual decision and outcome was applied (Selten, 1967). Table 2 depicts the table that subjects saw on their screens.

**Table 2:** Decision to increase or decrease the delegator’s pay-off

Lower or increase subjects’ payoff given that:	Amount
Subject used own work–outcome: success	*enter amount*
Subject used own work–outcome: failure	*enter amount*
Subject delegated (machine/human)—outcome: success	*enter amount*
Subject delegated (machine/human)—outcome: failure	*enter amount*

### Self-Assessment and Risk Attitudes

Subsequently, participants were asked to assess their own performance by estimating how many mistakes they had made during the logic task in the experiment’s first part. This estimate was incentivized by an additional payment of 50 ECU if they guessed correctly. This procedure allowed us to analyze the effect of self-assessments on delegation decisions. As is standard in incentivized economic experiments, the experiment was concluded by elicitation of participants’ risk attitudes.^1^

### Determination of Sample Size

The key dependent variable in our study is the relative reward (or punishment) for delegating the task versus using one’s own work in case of success and in case of failure. Because we elicit these relative rewards via the strategy method, we compare paired differences. After running a pilot, we performed an ad-hoc power analysis to determine the appropriate sample size per treatment. We calculated with an expected mean of the paired differences of 5.00 ECU, an expected standard deviation of the paired differences of 15.00, an error probability of *α* = 0.05 and a power of 1 − *β* = 0.8. These parameters determined a required sample size of 73 subjects for each of the two treatments.

## Results

The experiment was conducted at a major German university between February and May 2019. A total of 149 subjects participated in six sessions; 43% were female, and the average age was 23.08 years (SD = 3.83). Participants received a participation fee of 4.00 EUR and could earn additional money in the experiment. Each session lasted about 45 min and the average payment was about 13.50 EUR per participant. The experiment was programmed in z-Tree (Fischbacher, 2007), and subjects were recruited via ORSEE (Greiner et al., 2004). Data analysis was conducted using Python’s NumPy, SciPy and statsmodels.api libraries. The preprocessed data set and the code are available online.^2^

After the delegation decision, all participants were asked to evaluate the decision of the participant whose decisions had affected their own pay-off, as described in Section “Experiment Design”. The decision in question was to either delegate (to a human or machine) or rely on one’s own work (in both treatments). Note that participants were informed whether the participant they were evaluating had been assigned to the human or machine treatment but not whether the other participant had actually delegated or not. They were therefore asked to judge the decision with respect to the four possible outcomes according to the so-called strategy method (see Section “Experiment Design”). Subjects could increase (or decrease) the payment of their responsible participant by an integer between 0 and 40 for the four outcome combinations shown in Table 2: (1) own work results in positive outcome, (2) own work results in a negative outcome, (3) delegate’s work results in a positive outcome, and (4) delegate’s work results in a negative outcome. To test our conjecture that delegators are rewarded differently for delegating other-regarding tasks to artificial as opposed to human agents, we contrasted evaluations between both treatments.

Let us first consider the good-outcome case. In the human treatment, participants who did not delegate and caused a good outcome themselves were rewarded, on average, 20.28 ECU (SD = 20.85). Participants who delegated to another human who then brought about a good outcome on their behalf were rewarded 20.94 ECU (SD = 21.34). This difference is insignificant (*p* = 0.720, paired *t*-test).

In the machine treatment, participants who did not delegate and caused a good outcome themselves were rewarded, on average, 23.78 ECU (SD = 20.14). Participants who delegated to a machine that caused a good outcome were rewarded 27.00 ECU (SD = 16.71). This difference is again insignificant (*p* = 0.106, paired t-test).

### Result 1

 Delegators were rewarded equally for good outcomes that were caused by either human or artificial agents.

Let us now consider the bad-outcome case. In the human treatment, participants who did not delegate and caused a bad outcome themselves were rewarded, on average, 7.29 ECU (SD = 22.59 ECU). Participants who delegated to another human were rewarded, on average, 8.26 ECU (SD = 21.56 ECU). This difference is insignificant (*p* = 0.559, paired *t*-test).

In the machine treatment, participants who did not delegate and caused a bad outcome themselves were rewarded, on average, 8.53 ECU (SD = 26.52). Participants who delegated to a machine that caused a bad outcome were rewarded, on average, 12.96 ECU (SD = 24.44). This difference is significant (*p* = 0.041, paired *t*-test).

In the machine treatment, delegators earned higher rewards if a machine agent caused the failure. This confirms our conjecture stated in Section “Blame Avoidance”. Principals are rewarded differently for delegating tasks depending on the nature of the agent. More specifically, they fare better if a machine agent caused a bad outcome than if they had personally done it.

### Result 2

 Delegators did not effectively avoid punishment for a bad outcome if their human agent caused it instead of themselves. However, punishment was significantly lower if their artificial agent caused it instead of themselves.

**Fig. 3:**
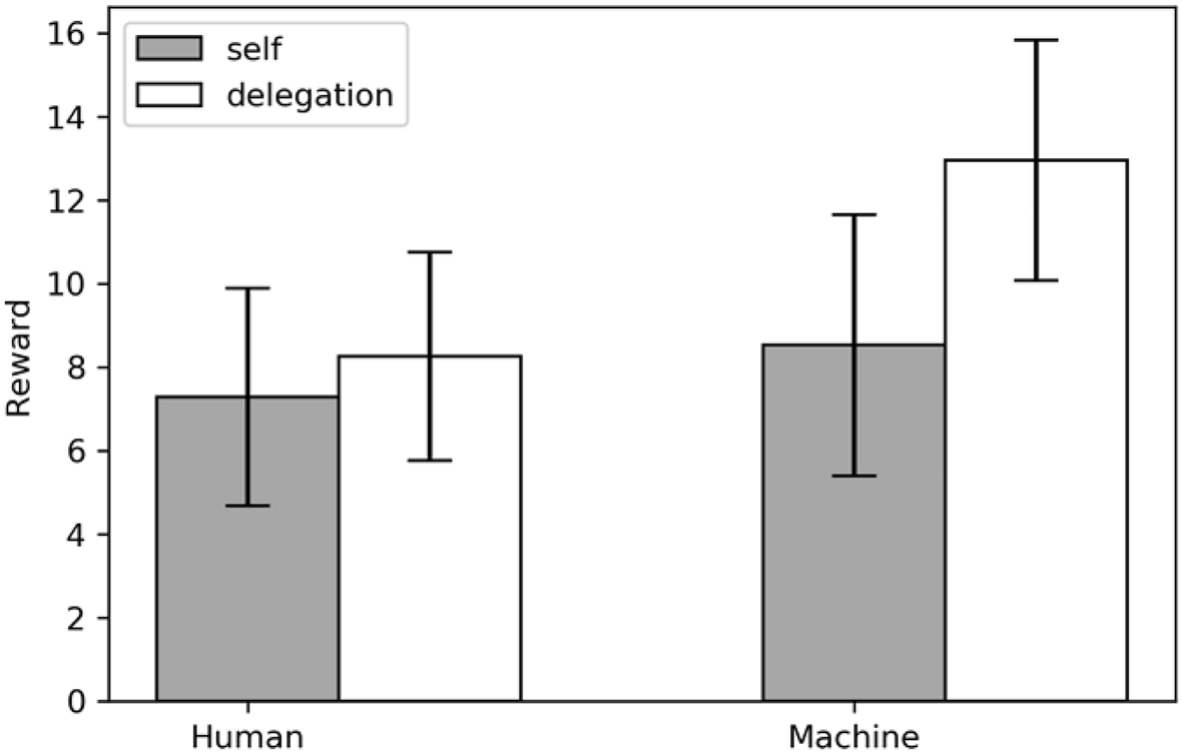
Reward decisions if the bad outcome prevails

Figure 3 illustrates this asymmetry in terms of rewards between the human and the machine treatment for the bad-outcome case.

To determine whether participants exploited this incentive to delegate to machine agents that did not exist for human agents, we compare the proportions of delegators in each treatment. In the human treatment, 35 out of 76 (46.1%) delegated the task to another human participant, whereas 41 out of 73 (56.2%) delegated to the machine agent in the machine treatment. The difference in the proportions of delegators is insignificant (*p* = 0.28, chi-square test for independence). The similar proportions of delegators in both treatments suggest that the decision to delegate to the agent was not primarily driven by strategic concerns of avoiding punishment in the event of a bad outcome.

This is corroborated by the logistic regression reported in Table 3. The propensity to delegate (1 = yes) is positively predicted by participants’ self-assessment of the number of errors they believed they had committed in the logic task (*p* < 0.001). “Machine” captures whether the agent is human (0) or artificial (1), which does not significantly influence this result. The degree to which self-assessment predicts the delegation decision is robust when controlled for risk attitude, although participants who were more risk averse were also more likely to delegate (*p* < 0.017). Note that subjects, on average, incorrectly solved 4.20 (SD = 2.33) puzzles out of ten, with an average of 3.90 (SD = 2.36) in the human treatment and 4.51 (SD = 2.27) in the machine treatment. The difference of incorrectly solved puzzles between the two treatments is insignificant (*p* = 0.117, independent *t*-test). It does therefore appear that the difficulty of the logic task was high enough to drive delegation while being consistent across the two treatments.

**Table 3:** Generalized linear model (binomial) regression results

Dep. Variable:	Del	No. Observations:	149
Model:	GLM	Df Residuals:	145
Model Family:	Binomial	Df Model:	3

	coef	std err	z	P $>|$ z $|$	[0.025	0.975]
Intercept	− 3.2254	0.795	− 4.055	0.000	− 4.784	− 1.666
Self-Assessment	0.4092	0.105	3.906	0.000	0.204	0.615
Machine	0.3228	0.365	0.885	0.376	− 0.392	1.038
Risk	0.1806	0.076	2.383	0.017	0.032	0.329

Thus, it appears that the expected utility of delegating was the driving factor behind participants’ decisions to do so or not, regardless of the nature of their agent. Those who were less confident regarding their own performance were more likely to delegate the task. Their estimated number of errors, which they stated in an incentivized self-assessment, had a significant impact on their delegation decision. As Table 4 shows, participants’ self-assessment is also predicted by the participants’ actual number of errors in the logic task. Whether they had human or artificial agents at their avail did not influence their self-assessment. Also, subjects’ risk attitudes had no impact on their self-assessment. These findings indicate that participants had realistic impressions of their performances. Subjects who performed better in the logic task were accordingly less likely to delegate.

**Table 4:** Linear regression–influences on self-assessment

Dep. Variable:	Self-Assessment	R-squared:	0.269
Model:	OLS	Adj. R-squared:	0.253
Method:	Least Squares	F-statistic:	17.74
No. Observations:	149		

	coef	std err	t	P $>|$ t $|$	[0.025	0.975]
Intercept	3.2012	0.474	6.749	0.000	2.264	4.139
Machine	0.4598	0.276	1.666	0.098	− 0.086	1.005
Error	0.3955	0.059	6.741	0.000	0.280	0.512
Risk	− 0.0298	0.055	− 0.539	0.591	− 0.139	0.079

## Conclusion

Our findings indicate that delegators may be judged with more leniency in the event of a bad outcome if they delegate tasks to artificial agents instead of human agents. It does, therefore, stand to reason that machine agents can be successfully used to avoid punishment. This has some troubling implications: Companies, for instance, might well capitalize on the effective shift of responsibility to algorithms if they fail. This is all the more true if they do not suffer any comparable loss of prestige for successful outcomes as a result of the delegation, as our data also suggest. The fact that the delegator in a between-subjects design receives a discharge if the agent is artificial but not if the agent is human suggests that the corresponding judgment is not based on ethical reflection but the result of subtle behavioral tendency.

Our results might indicate the importance of institutional solutions that hold companies, and ultimately individuals, liable for the harm that their artificial agents bring about. Such accountability measures are even more important if they have to compensate for consumers’ behavioral reluctance to attribute punishment in such cases. A deeper inquiry into the reluctance to punish that we observe seems warranted by the idea that extrinsic social motivation is an important factor in moral decision-making (Cappelen et al., 2017). The ability to use artificial agents to avoid accountability might encourage various decision-makers to engage in more activities that are considered undesirable by stakeholders. The reassuring fact that delegators in this experiment did not exploit the effective release from punishment does not imply that others will not–especially once this behavioral tendency is broadly recognized.

This is especially problematic in connection with the aforementioned retribution gap. The punishment patterns during our experiment indicate that people are, in fact, striving for corrective justice and are willing to re-allocate punishment when artificial agents bring about harm. As mentioned before, this can have real-life consequences regardless of one’s personal ethical stance, e.g., when people are angered by accidents involving autonomous vehicles. The issue becomes all the more pressing when we consider that delegators might start to deliberately use artificial agents to deflect punishment, thereby not dispelling anger but merely redirecting it.

Our experiment is subject to limitations. One is its rather explorative nature, which is owed to the lack of a well-established theoretical framework: Scientists have only recently begun to study empirical evidence on the influence of algorithms on human decision-making. The projects by Gogoll and Uhl (2018) and Strobel and Kirchkamp (2017) cited above are two of very few experiments dedicated to the issue so far. Furthermore, the stylized setting used in our experiment limits the scope of the conclusion that we can draw. This concern is best explained in relation to internal and external validity. Internal validity means that the experiment is designed in such a way that it warrants conclusions about the behavior of its participants inside of the laboratory, for instance, by keeping relevant factors constant (ceteris paribus) or omitting irrelevant influences (ceteris absentibus). Experiments are externally valid if their design produces findings that are informative about behavior outside of the laboratory (Guala, 2002). Some authors posit an inverse relationship between internal and external validity (Guala et al., 2005; Loewenstein, 1999).

For the task that we used, we could indeed identify a deflection of punishment if the task was delegated to a machine. In terms of internal validity, we were mainly concerned about similar error rates in the human and machine treatments to render the comparison between both treatments meaningful. In terms of external validity, however, it is conceivable that a task that is more or less difficult for humans would result in an interaction effect between error rates and the agent’s nature on the relative evaluation of the delegator. Correspondingly, our findings merely provide a first indication that the nature of the agent has an effect on the reward and punishment that the delegator receives. The phenomenon we observed would have to be replicated in other contexts inside and outside of the laboratory to eliminate the possibility of it being a mere artifact.

This is related to another issue that we have not addressed to this point – the behavioral effects of anthropomorphism on human–machine interaction. In this context, anthropomorphism describes the attribution of distinctively human characteristics to nonhuman entities, primarily a consciousness or conscious mind (Appel et al., 2020; Gray & Wegner, 2012; Gray et al., 2007; Shank, DeSanti, et al., 2019; Shank, Graves, et al., 2019). There is ample evidence for this attribution affecting peoples’ judgments and decision-making when interacting with machines. This includes increased trust in artificial agents (Bartneck et al., 2009; Waytz et al., 2014), a greater reluctance to sacrifice them (Nijssen et al., 2019), a higher willingness to hold them morally responsible for their actions (Malle & Scheutz, 2016), and even effects on blame attribution or perceived responsibility (Gray et al., 2011; Malle & Scheutz, 2016). Studies have also shown that the perception of the machine having a mind makes people willing to accept machines making moral decisions, namely in medical or military contexts (Bigman & Gray, 2018). However, we decided not to investigate this effect in our experiment. Various cultural, social, and situational factors influence people’s tendency to attribute human traits to non-human entities (Epley et al., 2007). Because we were unable to control for all these attributes, we decided to exclude this interesting component from our analysis. We, therefore, avoided triggers of anthropomorphization or perception of mind such as a humanoid depiction of the algorithm or assigning it a name, voice, or gender. Participants did also not interact with the algorithm directly. Instead, participants were given an accurate and truthful description of the algorithm and its capabilities, as described in Section “Experiment Design”.

Nevertheless, it seems warranted to conduct follow-up studies in which we compare reward and punishment patterns between treatments with our existing algorithm and a more humanized agent. We believe that the introduction of a more human-like agent could have strong effects on the results, partly because people are more willing to attribute guilt to an agent whose inner workings appear more similar to their own. Additionally, one might argue that the tendency to anthropomorphize non-human agents may be present regardless of the framing because it increases subjects’ perceived competence in understanding and interacting with them (Epley et al., 2007).

We believe our findings represent an early step in understanding delegation decisions in a domain that is gaining relevance, i.e., the use of artificial agents in moral decision-making. More generally, our experimental results illustrate the necessity of investigating the interaction between humans and machines in behavioral settings. It is insufficient to rely on ethicists’ armchair arguments and on surveys that study laymen’s intuitions regarding the ethical implications of algorithms because ethically relevant intuitions might arise as unintended results from the interactions between people and machines. The emerging phenomena might then be difficult to anticipate and sometimes even counter-intuitive. Further research is urgently needed to create a more conclusive picture of the subtle factors that drive our behavior when cooperating (and competing) with machines.

## Footnotes

1 We used the procedure introduced by Holt and Laury (2002). The task is based on ten choices between pairs of lotteries. The potential payoffs for the safe lottery range from 2.5 to 1.6 EUR and are therefore always less extreme than those for the risky lottery that range from 4.35 to 0.10 EUR. For the first pair of lotteries, the probability of the high payoff is equally low in both lotteries but equally increases for both lotteries with each new pair. A participant should switch to the risky lottery once the probability for the high payoff is sufficiently high according to his or her personal risk attitude. The later a participant switches to the risky lottery, the more risk averse he or she is.

2 https://doi.org/10.5281/zenodo.5078339.
